# A new scoring system derived from base excess and platelet count at presentation predicts mortality in paediatric meningococcal sepsis

**DOI:** 10.1186/cc12609

**Published:** 2013-04-11

**Authors:** Alexessander Couto-Alves, Victoria J Wright, Karnan Perumal, Alexander Binder, Enitan D Carrol, Marieke Emonts, Ronald de Groot, Jan Hazelzet, Taco Kuijpers, Simon Nadel, Werner Zenz, Padmanabhan Ramnarayan, Michael Levin, Lachlan Coin, David P Inwald

**Affiliations:** 1 Department of Epidemiology and Biostatistics, School of Public Health, Imperial College, Norfolk Place, London W2 1PG, UK; 2 Department of Paediatrics, Imperial College, Norfolk Place, London W2 1PG, UK; 3 Paediatric Intensive Care Unit, St Mary's Hospital, Imperial College Healthcare NHS Trust, Praed Street, London W2 1NY, UK; 4 Department of General Paediatrics, Medical University of Graz, Auenbruggerplatz 30, A-8036 Graz, Austria; 5 Institute of Child Health, University of Liverpool, Alder Hey Children's NHS Foundation Trust, Eaton Road, Liverpool L12 2AP, UK; 6 Department of Paediatrics, Division of Infectious Diseases and Immunology, Erasmus MC-Sophia Children's Hospital, Dr Molewaterplein 60, 3015 GJ, Rotterdam, Netherlands; 7 Radboud University Nijmegen Medical Centre, Geert Grooteplein 8, Route 463, 6500 HB Nijmegen, Netherlands; 8 Department of Paediatrics, Erasmus MC-Sophia Children's Hospital, Dr Molewaterplein 60, 3015 GJ, Rotterdam, Netherlands; 9 Department of Paediatric Haematology, Immunology and Infectious Diseases, Emma Children's Hospital, Academic Medical Centre, Meibergdreef 9, 1105 AZ Amsterdam, Netherlands; 10 Children's Acute Transport Service, PO Box 36829, London WC1N 3WH, UK

## Abstract

**Introduction:**

The aim of this study was to derive a novel prognostic score for mortality in paediatric meningococcal sepsis (MS) based on readily available laboratory markers.

**Methods:**

A multicentre retrospective cohort study for the consortium set and a single centre retrospective study for replication set. The consortium set were 1,073 children (age 1 week to 17.9 years) referred over a 15-year period (1996 to 2011), who had an admission diagnosis of MS, referred to paediatric intensive care units (PICUs) in six different European centres. The consortium set was split into a development set and validation set to derive the score. The replication set were 134 children with MS (age 2 weeks to 16 years) referred over a 4-year period (2007 to 2011) to PICUs via the Children's Acute Transport Service (CATS), London.

**Results:**

A total of 85/1,073 (7.9%) children in the consortium set died. A total of 16/134 (11.9%) children in the replication set died. Children dying in the consortium set had significantly lower base excess, C-reactive protein (CRP), platelet and white cell count, more deranged coagulation and higher lactate than survivors. Paediatric risk of mortality (PRISM) score, Glasgow meningococcal septicaemia prognosis score (GMSPS) and Rotterdam score were also higher. Using the consortium set, a new scoring system using base excess and platelet count at presentation, termed the BEP score, was mathematically developed and validated. BEP predicted mortality with high sensitivity and specificity scores (area under the curve (AUC) in the validation set = 0.86 and in the replication set = 0.96). In the validation set, BEP score performance (AUC = 0.86, confidence interval (CI): 0.80 to 0.91) was better than GMSPS (AUC = 0.77, CI: 0.68, 0.85), similar to Rotterdam (AUC = 0.87, CI: 0.81 to 0.93) and not as good as PRISM (AUC = 0.93, CI: 0.85 to 0.97).

**Conclusions:**

The BEP score, relying on only two variables that are quickly and objectively measurable and readily available at presentation, is highly sensitive and specific in predicting death from MS in childhood.

## Introduction

Despite vaccination against *Neisseria meningitidis *serogroup C, meningococcal sepsis (MS) with non-vaccine serogroups, particularly serogroup B, remains a major public health challenge. Meningococcal disease can evolve extremely rapidly, with non-specific symptoms evolving into severe sepsis with multiple organ failure within hours. Much attention has rightly been devoted to the development of an effective vaccine and to the education of the lay public and medical staff to help identify and act on symptoms of early disease. However in the UK, despite these measures, there are still around 1,000 cases of meningococcal sepsis every year, with about 200 children requiring intensive care. The case fatality rate is 5 to 10% and of those who survive, 10 to 20% develop permanent sequelae, including skin scars, limb amputation, hearing loss, seizures and brain damage [1,2].

A reliable prognostic scoring system would have two main purposes - first, to identify patients at high risk of deterioration, and second, for risk stratification in future trials of novel therapies in sepsis [3]. Previous clinical trials of specific novel therapies in meningococcal sepsis, targeting pathways of inflammation and coagulation such as recombinant bactericidal/permeability-increasing protein (rBPI) [4] and human activated protein C (rhAPC) [5], have failed for reasons which are not clear, but which may include case mix. Currently, therefore, therapy consists of antibiotics and supportive treatments only. Future trials of novel therapies in MS and indeed in other forms of bacterial sepsis are likely to succeed only if patients at high risk of severe disease are identified and enrolled, rather than patients reliably predicted to survive or die.

More than 20 previous studies have looked at factors which might be predictive of mortality in MS. These have included scoring systems which combine clinical data with laboratory data including the Glasgow meningococcal septicaemia prognostic score (GMSPS) [6] and the paediatric risk of mortality (PRISM) score [7], amongst numerous others (Table 1). The PRISM score, which is a general paediatric intensive care unit (PICU) severity of illness score, has been validated in MS [8]. Scoring systems solely based on laboratory markers include the product of the platelet and neutrophil count (the PN score) [9] and the Rotterdam score, based on potassium, base excess, platelet count and C-reactive protein (CRP) [10]. More recently, scores based on biomarkers have been proposed [11]. While biomarker scores can be highly accurate and are scientifically attractive, they are not useful at the point of presentation as the assays on which they depend take time to run. An ideal score would include a small number of variables that are quickly and objectively measurable, readily available at presentation and cost-effective.

**Table 1:** Data points used in the different scoring systems discussed in this paper.

Data point	PRISM	GMSPS	Rotterdam	BEP
Base deficit		√ (> 8 mmol/L)	√	√
HCO_3_	√			
Platelets			√	√
Potassium	√		√	
CRP			√	
Systolic BP	√	√ (< 75 mmHg)		
Diastolic BP	√			
GCS	√	√ (< 8)		
Heart rate	√			
Resp rate	√			
PaO_2_/FiO_2_	√			
PaCO_2_	√			
PT/PTT	√			
Bilirubin	√			
Calcium	√			
Glucose	√			
Pupil reaction	√			
Skin/rectal temp > 3 degrees		√		
Lack of meningism		√		
Parents opinion child is worse		√		
Ecchymoses		√		

Paediatric risk of mortality (PRISM), Glasgow meningococcal septicaemia prognosis score (GMSPS), Rotterdam score and base rate and platelet count (BEP) score. CRP, C-reactive protein; BP, blood pressure; GCS, Glasgow coma score; PT, prothrombin time: PTT, partial thromboplastin time.

Unfortunately, previous studies developing and testing such prognostic scoring systems have in the past not investigated large numbers of patients, the largest previous study looking at only 278 children [12]. Furthermore, PRISM, GMSPS and many of the other clinical or combined scoring systems depend to an extent on subjective assessments which may not be reliable. For example, GMSPS includes the variable 'parental opinion that child's condition has become worse over the past hour'. PRISM requires entry of multiple data points into a complex algorithm and thus is less attractive in emergency settings.

As part of a large European study of genetic factors contributing to disease severity and susceptibility in meningococcal disease, clinical and scoring data became available in a unique and substantial cohort of patients referred to PICUs in the United Kingdom, Austria, Germany and Holland [13].

The aim of this current study was to identify factors predictive of death in this population, including current scoring systems, when data was available. A new score was developed and validated using an independent cohort of patients referred to PICU via the North Thames regional retrieval service, the Children's Acute Transport Service, London, UK (CATS).

## Materials and methods

### Patients

Data was collected prospectively (and analysed retrospectively) from children referred to PICUs at participating centres over the 15-year study period, 1996 to 2011. Participating centres included St Mary's Hospital (London, UK), Alder Hey Children's Hospital (Liverpool, UK), Medical University of Graz (Graz, Austria), Erasmus-MC Sophia Children's Hospital (Rotterdam, Holland) Emma Children's Hospital (Amsterdam, Holland) (the 'consortium') and the Children's Acute Transport Service (CATS, London, UK). Clinical and other data in consortium patients were collected during ongoing studies at each hospital, approved by the institutional ethics committee for each participating centre. Informed consent was obtained for these patients according to local regulations. Retrospective data collection at CATS was approved by the Great Ormond Street Hospital for Children Clinical Audit Committee. Parental consent was deemed to be unnecessary for patients retrospectively included in this anonymised observational study. Each centre collected and recorded data into their own databases before entering anonymised data onto a centralised web-based data collection system (Dataphiles, Otley, UK).

### Definitions

Any child referred to PICU with a clinical diagnosis of MS was eligible for inclusion. The clinical diagnosis of MS required the presence of fever and haemorrhagic rash together with clinical features of severe sepsis or septic shock, according to the criteria described by Goldstein *et al. *[14]. Children with isolated meningitis without sepsis physiology were excluded from the analysis.

### Data

Anonymised data exports from the participating centres' databases were sent and uploaded into a central database held at Imperial College London for further analysis. Data included in the anonymised export were demographic data (age, sex, admission date, ethnic origin), diagnostic data (sepsis or meningitis as main presenting feature, serogroup of organism if available), clinical scores (PRISM, GMSPS and Rotterdam score), laboratory data (platelet count, white cell count, lactate, base excess coagulation profile, fibrinogen, potassium, CRP) and outcome data (survival). Unfortunately, outcomes other than death, including important morbidities such as skin loss requiring grafting or limb amputation, were not reliably recorded and hence could not be investigated in this study. Laboratory data, from the first recorded sample, were measured in each centre according to standard techniques.

### Statistical analysis

Differences within the study groups were analysed with the *t*-test for unequal variance. Robust estimation of the area under the curve (AUC) of the receiver operating characteristic (ROC) was computed and its 95% confidence interval (CI) was estimated using a bootstrap method implemented in the R statistical package [15]. The statistical significance of the AUC was assessed using the Mann-Whitney test. The best cutoff point according to the Youden's statistic was defined as previously described [16]. Calibration of the model was assessed using Cox's calibration regression in the R statistical package.

#### Development and validation datasets

**Figure 1:**
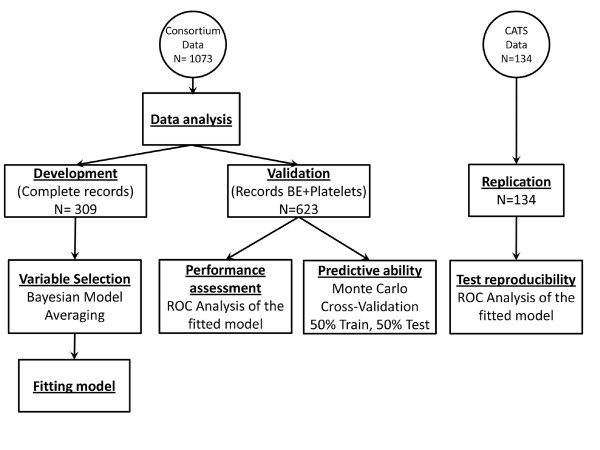
**Methodology**. Data from two sources were collected: data contributed by consortium members (development and validation set) and by the Children's Acute Transport Service (replication set). Development set is subdivided into two sets. The complete records set includes those with complete information for all variables collected. Validation records are those records that include complete information for base excess (BE) and platelets but are not all variables.

All records in the consortium set containing complete information on all laboratory variables plus gender were used as the development set (*n *= 309). The remainder of the records was used as the validation set (*n *= 623) (see Figure 1).

#### Variable selection and survival prognosis score

Bayesian model averaging (BMA) for generalised linear models [17] was applied to the development set to identify variables significantly (α = 0.05) associated with survival outcome [15]. All available laboratory variables were included in this analysis (Figure S1 in Additional file 1). Logistic regression analysis was conducted to determine whether other study variables, including study centre, year of admission, age, weight and sex were associated with death. The new prognosis score was based on a logistic regression model developed using the selected laboratory variables and fit to the development set.

#### Survival prognosis score assessment on the validation dataset

The new prognosis score was assessed in the validation dataset and ROC curves as well as the AUC were compared with other benchmark prognosis scores from the literature using a bootstrap test for two paired ROC curves [18]. Monte Carlo cross validation was used to assess the performance of the logistic regression model on unseen data [19]. In the Monte Carlo analysis, data of the validation set was randomly split into equal train and test sets and the regression model was fit to the train set and evaluated on the test set (Figure 1). On each test set randomly generated, a performance statistic based on AUC test statistics described by DeLong *et al. *[20,21] was computed for the paired comparison between the new score and the benchmark score. This procedure was repeated 10^5 ^times to obtain a distribution of the performance statistic in the validation set.

#### Replication set

A ROC curve using the model was produced from a recent replication dataset of 134 patients referred to CATS, comprising 16 non-survivors and 116 survivors.

## Results

A total of 1,073 children (age 1 week to 17.9 years) with meningococcal sepsis were in the consortium set. Of these, 85 (8%) died; 581 (54%) were male. Demographics, clinical scores, laboratory data, interventions and outcome data are shown in Table 2. Eight children had Group A disease, 419 had Group B, 215 had Group C, 8 had W135, 4 were YW135 and 13 were logged as having positive cultures but no serogroup was available. The histograms of the laboratory data are shown in Figure S1 in Additional file 1. A total of 134 children (age 2 weeks to 16 years) were in the CATS replication set, of whom 16 (12% died). Details of the replication set are shown in Table 3.

**Table 2:** Demographic and other features of the study population, according to survival (mean with 95% confidence intervals shown, *t*-test with unequal variance).

	Whole population	Died	Survived	*P *value
Sex (male, %)	581/1073 (54%)	52/85 (61%)	529/988 (54%)	0.18 (chi square)
Decimal age (years)	5.12 ± 0.30	5.75 ± 1.3	5.06 ± 0.3	0.36
PRISM	12.7 ± 1.2	61.9 ± 6.7	11.0 ± 1.3	< 0.001
GMSPS	7.9 ± 0.2	11.4 ±	7.6 ± 0.3	< 0.001
Rotterdam	13.5 ± 1.6	59.1 ± 8.3	10.2 ± 1.3	< 0.001
WCC (10^9^/L)	14.5 ± 1.3	6.7 ± 1.5	15.1 ± 1.4	< 0.001
Platelets (10^12^/L)	197 ± 7	100 ± 19	206 ± 6.7	< 0.001
APTT (s)	53 ± 2	104 ± 15	50 ± 2	< 0.001
INR	1.7 ± 0.05	2.7 ± 0.4	1.6 ± 0	< 0.001
Fibrinogen (g/L)	3.7 ± 0.1	1.6 ± 0.4	3.9 ± 0.1	< 0.001
CRP (mg/dL)	107.3 ± 4.8	68.7 ± 12.1	110.1 ± 5	< 0.001
K (mmol/L)	3.7 ± 0	3.9 ± 0.3	3.6 ± 0	0.56
Base excess (mmol/L)	-6.9 ± 0.3	-12.1 ± 1.2	-6.5 ± 0.3	< 0.001
Lactate (mmol/L)	3.7 ± 0.2	7.2 ± 0.9	3.7 ± 0.2	< 0.001
Ventilation	627/1073 (58%)	80/85 (94%)	547/988 (55%)	< 0.0001 (chi square)
Inotropes	675/1073 (63%)	78/85 (96%)	597/988 (60%)	< 0.0001 (chi square)

PRISM, paediatric risk of mortality; GMSPS, Glasgow meningococcal septicaemia prognosis score; WCC, white cell count; APTT, activated partial thromboplastin time; INR, international normalised ratio; CRP, C-reactive protein.

**Table 3:** Details of replication set from the Children's Acute Transport Service, according to survival (mean with 95% confidence interval shown, *t*-test with unequal variance).

	Whole population	Died	Survived	*P *value
Sex (male, %)	79/134 (59%)	7/14 (50%)	72/120 (60%)	0.47 (chi square)
Decimal age (years)	3.25 ± 0.59	2.48 ± 0.52	3.35 ± 0.6	0.31
Platelets (10^12^/L)	205 ± 20	46 ± 8	227 ± 19	< 0.001
Base excess (mmol/L)	-7.4 ± 0.8	-15.4 ± 1.0	-6.3 ± 0.6	< 0.001

### Development of a new score

A model built using BMA for logistic regression with binomial distribution prior probability (*P *= 0.5) of inclusion of a variable was fitted to the records of the development dataset with complete information for all variables (*n *= 309). The posterior probability of the model coefficients was analysed to identify variables to be included in the new prognosis score. Supplementary Figure S2 shows the posterior probability distribution of each variable coefficient (Figure S2 in Additional file 2). The variables that were most statistically important (α = 0.05), base excess (BE) and platelets, were used to build a model using logistic regression, termed the BEP score. The fitted model was statistically significant (likelihood ratio test *P *< 0.001, Hosmer-Lemeshow chi-square test = 20.2, *P *= 0.009) with considerable goodness of fit (Nagelkerke pseudo-R^2 ^= 0.3 and Brier score = 0.046). Calibration of the BEP score on the entire consortium dataset shows a relatively small underestimation of the probability of death for BEP > 0.3 (the mean absolute error is 0.025 and the 0.9 quantile of the absolute error is only 0.066, (Figure S3 in Additional file 3). We tested for confounding or study design effects and did not find any association with study centre, year of admission, age, weight or sex (*P *> 0.6 for all variables).

### BEP score regression coefficient analysis

We estimated the coefficients of the logistic regression model in the entire development dataset. Overall, the statistical significance of the variables is high and standard error of the coefficients is low, indicating that the choice of variables used in the model is appropriate and that the values of the coefficients are robust (data not shown). Using this analysis, the BEP score is mathematically defined as:

$$\text{P}\left(\text{death}\right)\;=\;\text{1}/\left(\text{1 }+\;{\text{e}}^{\left(0.18909\;\times BE\right)\;+\;\left(0.01015\;\times Platelets\right)\;+\;3.07861}\right)$$

### BEP score cutoff

**Figure 2:**
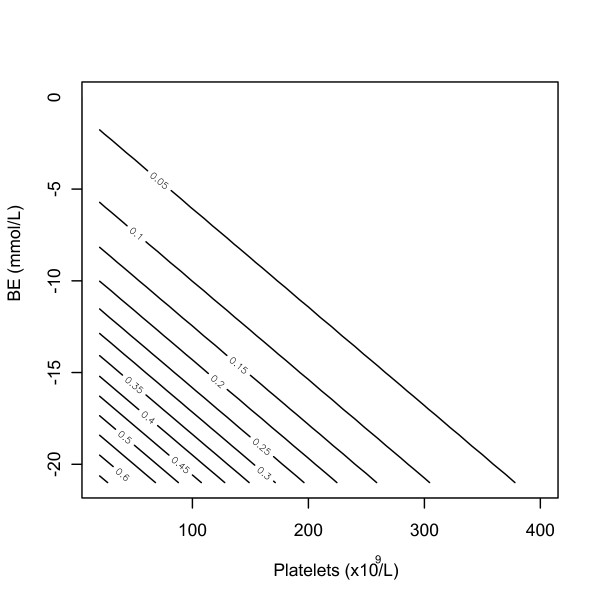
**Contour plot showing the probability of death for different ranges of base excess and platelet count (BEP) score**. Notice for BEP > 0.15, the probability of death increases progressively for the same change in the variables.

The performance of the BEP score for a range of cutoffs ((0.1 to 0.5)) was estimated on the development, validation and replication datasets (Table 4). For a cutoff as low as BEP > 0.3 a good discriminating performance can be achieved on all datasets positive predictive value ((PPV) > 0.5, negative predictive value (NPV) > 0.94). A contour plot of the BEP score probability of death as a function of BE and platelet count is shown for quick reference (Figure 2). The cutoff Θ that maximizes the Youden's statistic was estimated on the development dataset using the following equation: Θ = arg max (Sensitivity (Θ) + Specificity (Θ) -1). The cutoff was then applied to the validation and replication dataset and results are presented in Table S1 (Table S1 in Additional file 4). Overall, a good performance was consistently obtained in the validation and development datasets. Sensitivity confidence intervals obtained in the validation dataset includes the point estimates of the development dataset. Specificity confidence intervals obtained in development and validation datasets overlap and PPV was higher in the validation and replication dataset.

**Table 4:** Base excess and platelet count (BEP) performance for different cutoffs on the development, validation and replication datasets.

Dataset	BEP	N	Sensitivity	Specificity	Positive predictive value	Negative predictive value
	> 0.1	50	0.65	0.88	0.29	0.97
	> 0.2	21	0.44	0.96	0.45	0.96
Development	> 0.3	8	0.26	0.99	0.6	0.94
	> 0.4	4	0.15	1	0.75	0.94
	> 0.5	2	0.05	1	0.55	0.93

	> 0.1	110	0.67	0.87	0.29	0.97
	> 0.2	64	0.57	0.94	0.43	0.96
Validation	> 0.3	30	0.35	0.98	0.55	0.95
	> 0.4	21	0.29	0.99	0.68	0.95
	> 0.5	11	0.17	1	0.78	0.94

	> 0.1	25	0.85	0.9	0.4	0.99
	> 0.2	16	0.8	0.97	0.7	0.98
Replication	> 0.3	11	0.56	0.98	0.72	0.97
	> 0.4	9	0.48	0.99	0.79	0.96
	> 0.5	6	0.36	1	1	0.95

N denotes the number of individuals with BEP score greater than the selected cutoff (for example on the validation dataset there are 110 subjects with a BEP > 0.1).

### Performance analysis of the scores on the validation data

**Figure 3:**
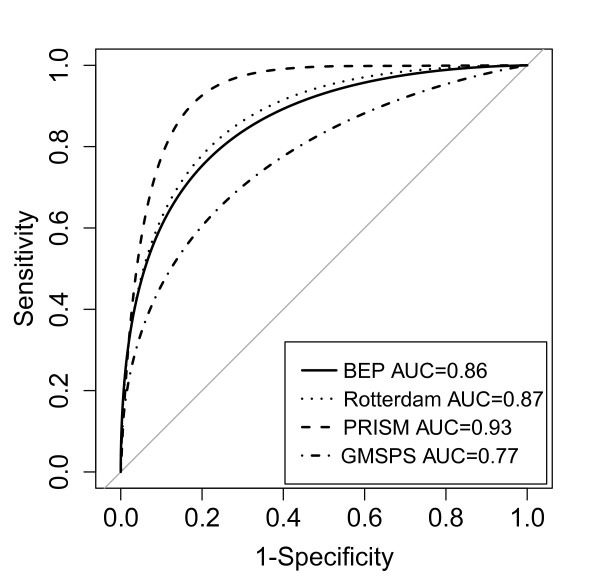
**Receiver operating characteristic (ROC) curve of each score on the validation set (*n *= 623)**.

The validation dataset was used to compare the performance of the different scores. These data consists of 623 individuals, 51 non-survivors and 572 survivors. ROC curves were generated for each score (Figure 3). BEP score performance (AUC = 0.86, CI: 0.80 to 0.91) was better than GMSPS (AUC = 0.77, CI: 0.68, 0.85), followed by Rotterdam (AUC = 0.87, CI: 0.81 to 0.93) and PRISM (AUC = 0.93, CI: 0.85 to 0.97). The bootstrap test for paired ROC curves demonstrated that BEP score was statistically significantly different from GMSPS (*P *= 0.03) but not significantly different from Rotterdam and PRISM (*P *= 0.68 and *P *= 0.22 respectively). The Monte Carlo cross validation analysis of the entire validation set (*n *= 623) demonstrated similar results (Figure S4 in Additional file 5), with BEP score AUC significantly different to PRISM (*P *= 0.04), almost significantly different to GMSPS (*P *= 0.056) but not different to Rotterdam (*P *= 0.541). Taken together, these results suggest that the BEP score is more accurate than GMSPS, equivalent to Rotterdam and not quite as accurate as PRISM.

### Replication of the BEP score performance on out-of-sample data

**Figure 4:**
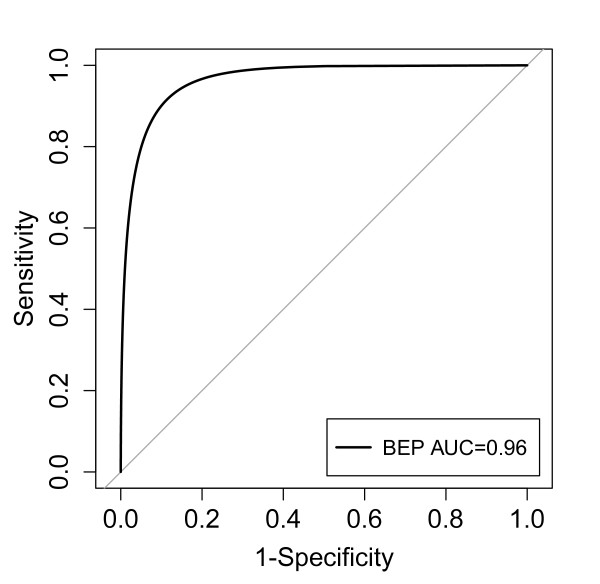
**Receiver operating characteristic (ROC) curve of the base excess and platelet count (BEP) score on the replication dataset (*n *= 134)**.

A replication dataset of 134 additional records from CATS were collected comprising 16 non-survivors and 116 survivors. The performance of the BEP score was evaluated using AUC and the ROC curve (Figure 4). The AUC on the replication dataset (AUC = 0.96, CI: 0.90 to 0.99).

## Discussion

Numerous studies have looked at factors predictive of poor outcome in meningococcal disease, since the seminal study of Stiehm and Damrosch in 1966, which identified petechiae, hypotension, absence of meningitis, peripheral leukopenia and low erythrocyte sedimentation rate (ESR) as markers of a poor prognosis [22]. Since then, in the developed world, the case fatality rate from meningococcal disease has fallen from over 50% to under 10%, largely due to improvements in diagnosis and supportive therapy [23,24]. However, trials of specific adjunctive therapies have not demonstrated any benefit. One reason for this may have been a failure to select a study population in whom neither death nor survival was inevitable. Therefore, any scoring system which helps to identify such populations may be of benefit in future clinical trials. Identification of populations of patients at high risk of death may also be useful for genetic studies looking at associations between genotype and disease severity.

Early studies developing prognostic scores in meningococcal sepsis tended to focus on clinical factors indicative of shock, which are amenable to supportive treatments, such as fluid loading, inotropic support and mechanical ventilation. One relatively recent study looking at laboratory markers focused on platelet and neutrophil count [8], and suggested that in an age of improved recognition and management of disease, markers of endothelial dysfunction were more useful than clinical markers of poor perfusion, the former representing pathophysiology not amenable to any specific treatment. However, GMSPS and PRISM, and scoring systems which combine clinical and laboratory markers continue to have clinical utility [25,26]. Clearly, an accurate scoring system using a small number of variables which are rapidly available soon after the patient comes through the doors of the Emergency Department will have more utility than a score dependent on multiple variables, some of which may be difficult or slow to measure, require repeated measurement or which rely on subjective assessment.

In this study, the largest to date in meningococcal sepsis, a new and extremely simple scoring system is proposed. This new score, termed the BEP score, is solely based on base excess and platelet count, both very easy to measure variables which represent tissue hypoperfusion and endothelial damage respectively. With an AUC of 0.86 in the validation set and 0.96 in the replication set, BEP performed significantly better than GMSPS, similarly to Rotterdam score, and not as well as PRISM. BEP also performed similarly to PN product in its original description looking at patients admitted prior to 1999, in which PN product was demonstrated to have an AUC of 0.97 in the development set and 0.89 in the validation set [9]. Unfortunately, we were unable to look at the PN score in our patients as absolute neutrophil count was not consistently available in our dataset.

In our population PRISM had an AUC of 0.93, having previously been described as anywhere between 0.80 [26] to 0.95 [8]. This indicates that despite being developed 25 years ago, PRISM remains a reliable score. Its main disadvantages are that it requires collection and entry of multiple data points into a complex algorithm and that it has not been validated in a pre-PICU setting. GMSPS, which has some constituent variables which are notoriously difficult to measure objectively, has been reported to have an AUC from 0.96 [12] to 0.81 [9] in previous studies, with a tendency to decrease from the 1980s to the present day. In our population GMSPS had an AUC of 0.77. This continued fall in the performance of GMSPS may be because of ongoing improvements in disease recognition and in initiation of supportive treatments, or other as yet unidentified factors. The Rotterdam score had intermediate reliability in our validation set, with an AUC of 0.87. Unfortunately, there is no historical AUC to compare with Rotterdam score, AUC being unreported in the initial study describing the score [10].

### Limitations

The most important limitation of this study is the fact that 'first recorded sample' is not clearly defined, the data consisting of multiple datasets from several European countries. While in some datasets the first recorded sample is that taken on presentation in the Emergency Department, in others the first sample was from the ICU admission. However, given the natural history of meningococcal disease, these time points are unlikely to have been more than a few hours apart. Additionally, the type of blood gas sample (whether arterial, capillary or venous), the calculation used to calculate base excess by the blood gas analyser and the resuscitation fluid administered were not recorded in the database.

A further limitation is that a few early deaths may have been missed out from the consortium set, that is, deaths occurring at referring hospitals prior to PICU referral. However the excellent performance of the BEP score despite this heterogeneity may also be considered to be a strength. Furthermore the fact that the score performed so well in the more homogeneous replication set, in which the first recorded sample was from presentation in Emergency Department and in which early deaths were likely to have been more reliably recorded, suggests that the score may have high utility at this important time point in the natural history of the disease.

Unfortunately, it was not possible to compare the BEP score to some previously described clinical scoring systems in this study because the data items used in those scoring systems were not available. Therefore it is still possible that another system based on clinical or other data might outperform the BEP score. Furthermore, while it would have been interesting to look at variations in performance of the BEP score over time, or across different centres or countries, it was not possible to do this given the nature of the data as some centres contributed more patients in the early part of the study period and others later. Hence it was not possible to dissect the individual contribution of these factors.

We were also unable to compare the performance of the BEP score to any other score in the replication dataset from CATS, as the data needed to produce these scores was not available retrospectively. It is also unclear as to why the BEP score had a higher AUC in the CATS replication dataset as compared to the validation dataset, although this may be due to the more homogeneous cohort which will have reliably included children who died in the referring centre prior to PICU admission.

## Conclusions

In this study, the development of a new prognostic score for meningococcal sepsis is described. The new score, termed the BEP score, depends on base excess and platelet count at presentation. Both of these variables are objective and easy and quick to measure. They are also unlikely to be affected by observer error. We propose that the BEP score should be further evaluated for mortality prediction and risk stratification in meningococcal and in other forms of bacterial sepsis, in both adults and children.

## Key messages

• A new prognostic scoring system for paediatric meningococcal sepsis was developed and validated in a cohort of 1,073 patients and tested in a replication set of 134 patients.

• The score, based on base excess and platelet count at presentation, has been named the BEP score

• Both base excess and platelet count are objective and easy to measure

• The BEP score is both sensitive and specific in predicting death

• Further evaluation is required to determine the utility of the BEP score in other forms of sepsis

## Abbreviations

AUC: area under the curve; BEP: base excess platelet count score; BMA: Bayesian model averaging; CI: confidence interval; CRP: C-reactive protein; ESR: erythrocyte sedimentation rate; GMSPS: Glasgow meningococcal septicaemia prognosis score; MS: meningococcal sepsis; NPV: negative predictive value; PICU: paediatric intensive care unit; PN: platelet-neutrophil product; PPV: positive predictive value; PRISM: paediatric risk of mortality; rhAPC: recombinant human activated protein C; rBPI: recombinant bactericidal/permeability-increasing protein; ROC: receiver operating curve.

## Competing interests

The authors declare that they have no competing interests.

## Authors' contributions

ACA carried out the statistical analysis, interpreted the data and drafted the manuscript. LC advised on the statistical analysis and helped to draft the manuscript. DI conceived of the study, and participated in its design and coordination, analysed and interpreted the data and drafted the manuscript. VW, KP, AB, EC, ME, RdG, JH, TK, SN, WZ and PR acquired data for the study and approved the final manuscript. ML contributed to conception and design and approved the final manuscript. All authors read and approved the final manuscript.

## Supplementary Material

Additional file 1 **Figure S1: Histograms of the laboratory variables**. Click here for file

Additional file 2 **Figure S2: Posterior probability distribution of the regression coefficients estimates using Bayesian model averaging**. Click here for file

Additional file 3 **Figure S3: Calibration curves showing agreement between predicted and observed probability of death**. Click here for file

Additional file 4 **Table S1: Base excess and platelet count (BEP) score cutoff values that optimise the Youden Index and associated performance**. Cutoff was estimated on development dataset and tested on all other sets. PPV, positive predictive value. Click here for file

Additional file 5 **Figure S4: Monte-Carlo cross validation estimate of the out of sample area under the curve (AUC) for base excess and platelet count (BEP) and all benchmark scores using 10^5 ^random splits of the validation set into two equal sets for training and testing BEP score**. **(A-C) **Z-score of the DeLong test statistics for the paired comparison between BEP and all benchmark prognosis scores. **(D-F) **Histograms of the AUC for each benchmark score. **(G-H) **Histograms of the BEP AUC on the set of records in common with each benchmark score. Click here for file
