# Supplementary figures and images for: A new scoring system derived from base excess and platelet count at presentation predicts mortality in paediatric meningococcal sepsis
Source: Crit Care. 2013 Apr 11;17(2):R68. doi: 10.1186/cc12609 (PMC3672696; doi:10.1186/cc12609)

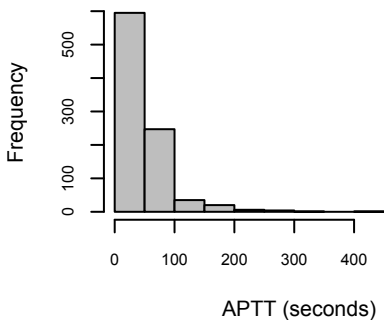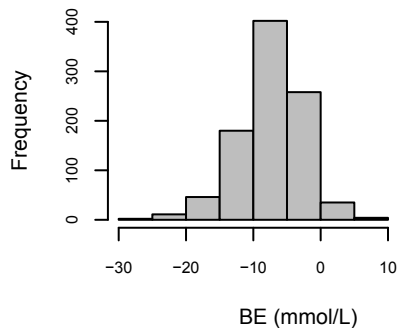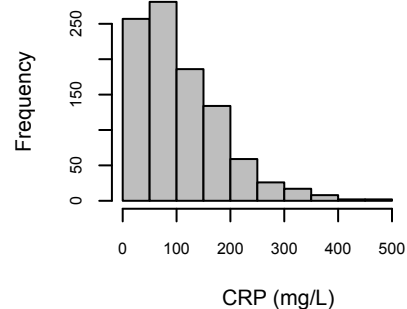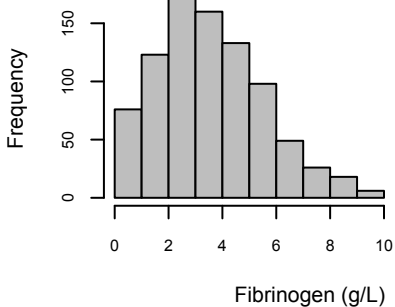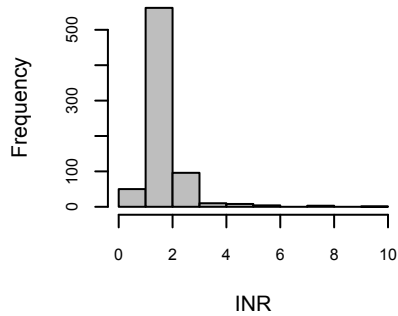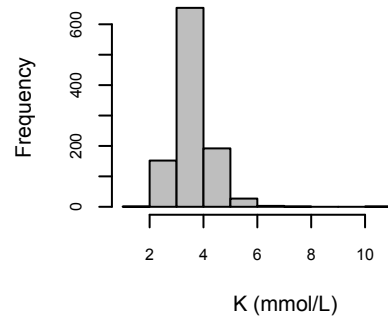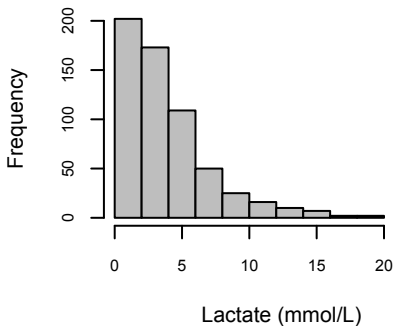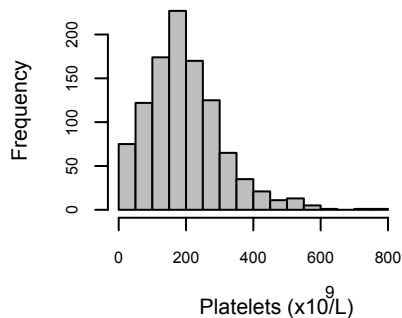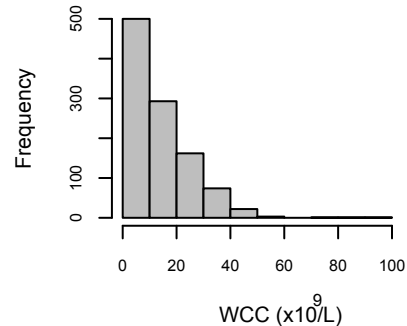

Supplement: Additional file 1 — Figure S1: Histograms of the laboratory variables. [file cc12609-S1.PDF]

**APTT**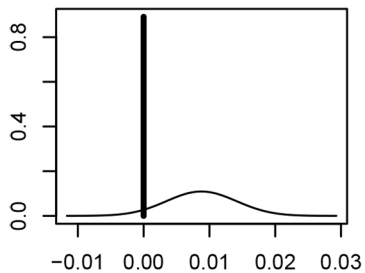**BE**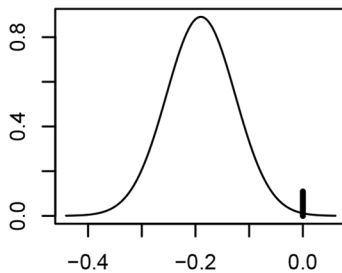**CRP**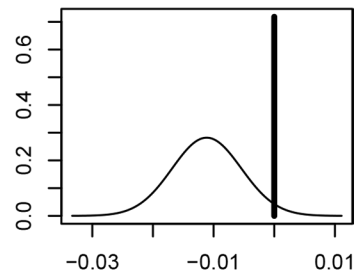**Fib**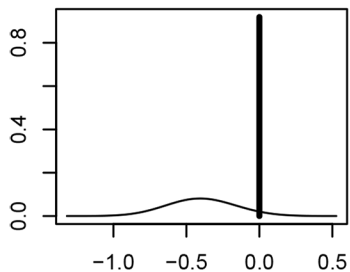**INR**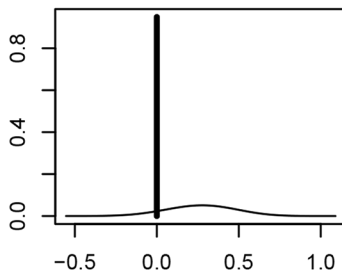**K**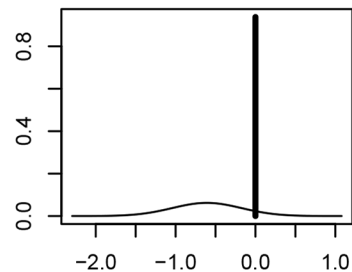**Lactate**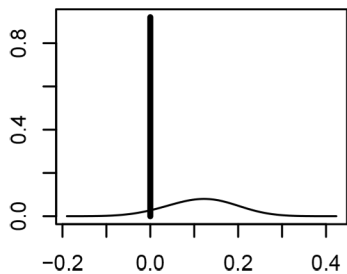**Platelets**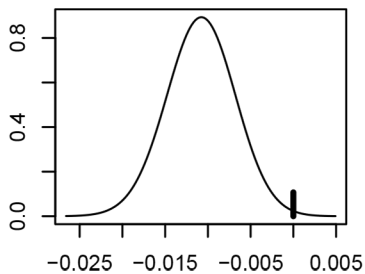**WCC**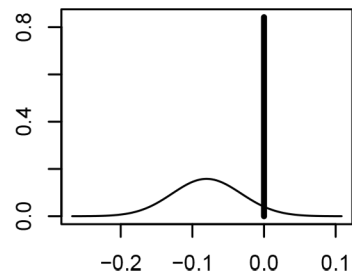

Supplement: Additional file 2 — Figure S2: Posterior probability distribution of the regression coefficients estimates using Bayesian model averaging. [file cc12609-S2.PDF]

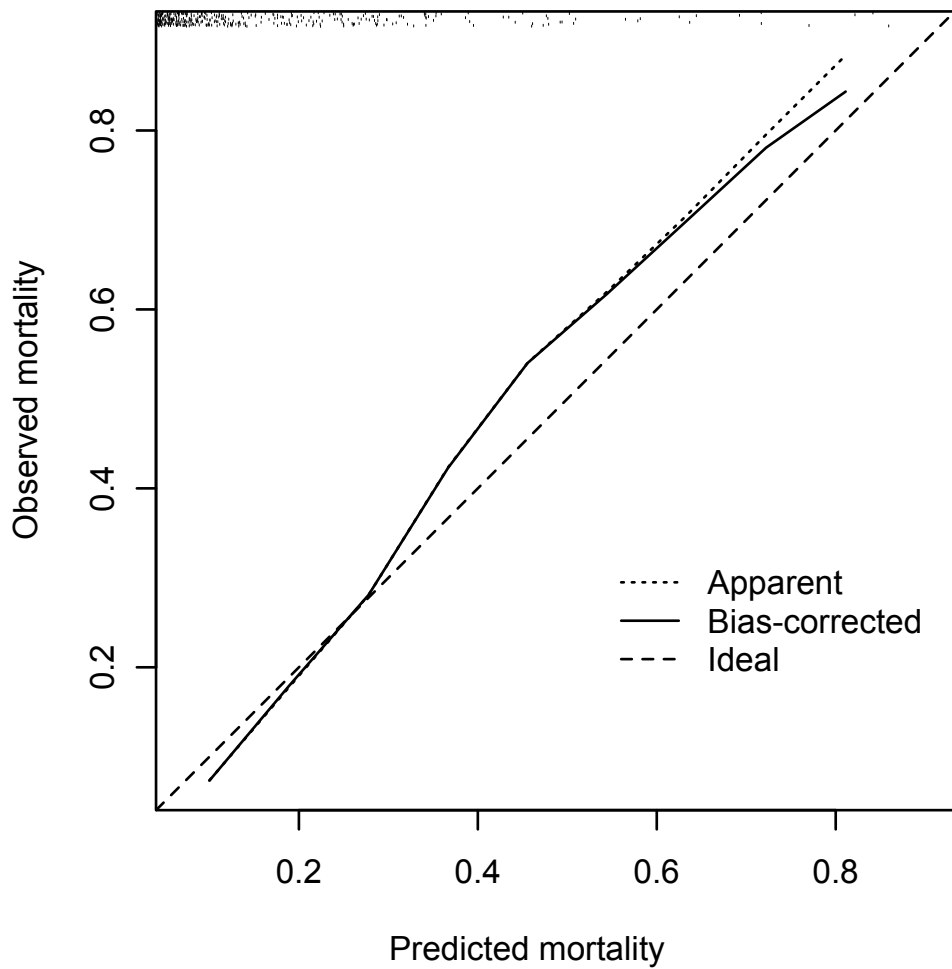

Supplement: Additional file 3 — Figure S3: Calibration curves showing agreement between predicted and observed probability of death. [file cc12609-S3.PDF]

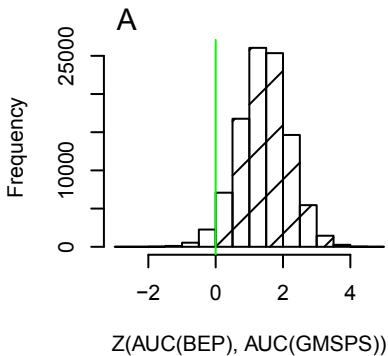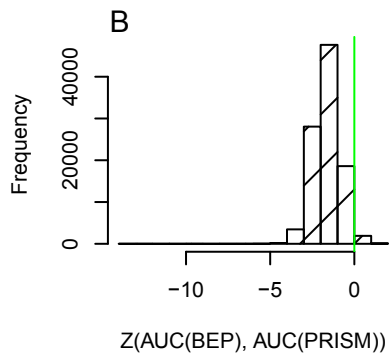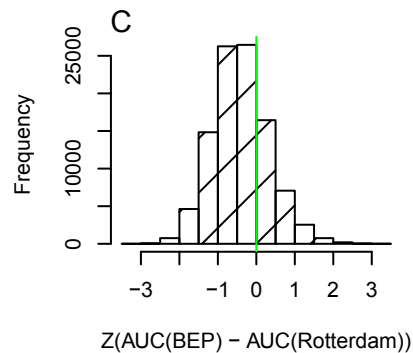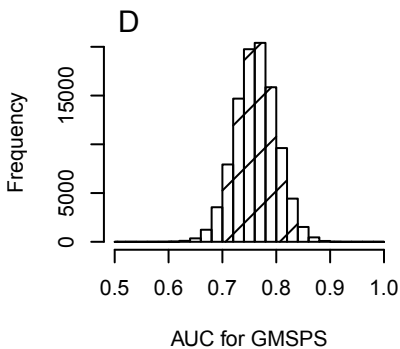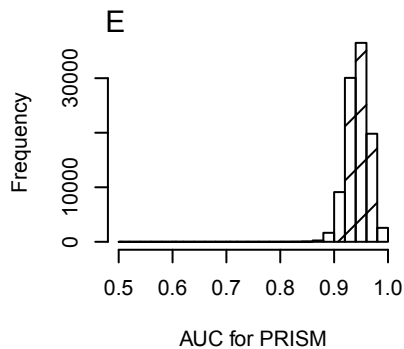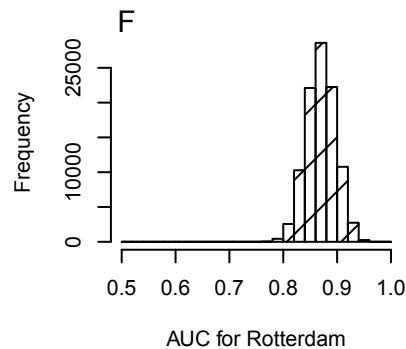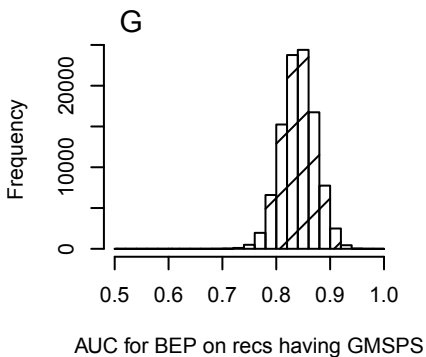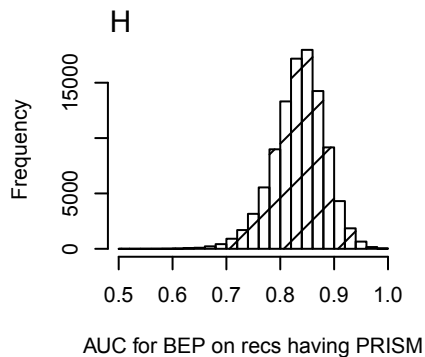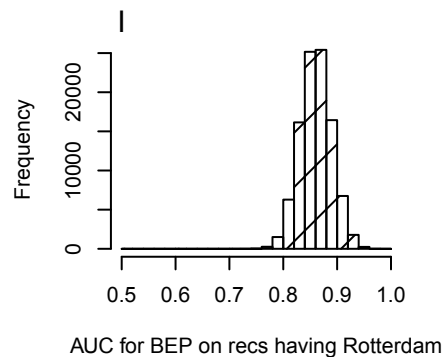

Supplement: Additional file 5 — Figure S4: Monte-Carlo cross validation estimate of the out of sample area under the curve (AUC) for base excess and platelet count (BEP) and all benchmark scores using 105 random splits of the validation set into two equal sets for training and testing BEP score. (A-C) Z-score of the DeLong test statistics for the paired comparison between BEP and all benchmark prognosis scores. (D-F) Histograms of the AUC for each benchmark score. (G-H) Histograms of the BEP AUC on the set of records in common with each benchmark score. [file cc12609-S5.PDF]
