# Supplementary material for: A new scoring system derived from base excess and platelet count at presentation predicts mortality in paediatric meningococcal sepsis
Source: Crit Care. 2013 Apr 11;17(2):R68. doi: 10.1186/cc12609 (PMC3672696; doi:10.1186/cc12609)
Supplement: Additional file 4 — Table S1: Base excess and platelet count (BEP) score cutoff values that optimise the Youden Index and associated performance. Cutoff was estimated on development dataset and tested on all other sets. PPV, positive predictive value. [file cc12609-S4.DOC]

**Table S1**

|  |  |  | Bootstrap 95% confidence interval | |
| --- | --- | --- | --- | --- |
| Dataset |  |  | Lower bound | Upper bound |
| Development | Sensitivity | 0.78 | 0.6 | 1 |
|  | Specificity | 0.83 | 0.57 | 0.96 |
|  | PPV | 0.22 | 0.12 | 0.51 |
|  | Cut-off | 0.075 | 0.031 | 0.177 |
|  | Youden's J | 0.61 | 0.47 | 0.82 |
| Validation | Sensitivity | 0.72 | 0.6 | 0.85 |
|  | Specificity | 0.82 | 0.79 | 0.85 |
|  | PPV | 0.23 | 0.16 | 0.25 |
|  | Cut-off | 0.075 | 0.0742 | 0.0752 |
|  | Youden's J | 0.55 | 0.4 | 0.67 |
| Replication | Sensitivity | 1 | 1 | 1 |
|  | Specificity | 0.87 | 0.8 | 0.93 |
|  | PPV | 0.52 | 0.42 | 0.67 |
|  | Cut-off | 0.073 | 0.07 | 0.081 |
|  | Youden's J | 0.88 | 0.8 | 0.93 |
